# Neuromedin U, a Key Molecule in Metabolic Disorders

**DOI:** 10.3390/ijms22084238

**Published:** 2021-04-19

**Authors:** Hitoshi Teranishi, Reiko Hanada

**Affiliations:** Department of Neurophysiology, Faculty of Medicine, Oita University, Oita 870-5593, Japan; teranishi@oita-u.ac.jp

**Keywords:** neuropeptide, NMU system, feeding behavior, energy expenditure, obesity, brain reward system, insulin secretion, inflammation

## Abstract

Obesity is now a public health concern. The leading cause of obesity is an energy imbalance between ingested and expended calories. The mechanisms of feeding behavior and energy metabolism are regulated by a complex of various kinds of molecules, including anorexigenic and orexigenic neuropeptides. One of these neuropeptides, neuromedin U (NMU), was isolated in the 1980s, and its specific receptors, NMUR1 and NMUR2, were defined in 2000. A series of subsequent studies has revealed many of the physiological roles of the NMU system, including in feeding behavior, energy expenditure, stress responses, circadian rhythmicity, and inflammation. Particularly over the past decades, many reports have indicated that the NMU system plays an essential and direct role in regulating body weight, feeding behavior, energy metabolism, and insulin secretion, which are tightly linked to obesity pathophysiology. Furthermore, another ligand of NMU receptors, NMS (neuromedin S), was identified in 2005. NMS has physiological functions similar to those of NMU. This review summarizes recent observations of the NMU system in relation to the pathophysiology of obesity in both the central nervous systems and the peripheral tissues.

## 1. Introduction

Obesity is a global epidemic caused by an imbalance resulting from excessive energy intake and inefficient energy expenditure [1,2]. Feeding behavior and energy homeostasis are regulated in a complex manner by various kinds of appetite-regulating peptides, including orexigenic neuropeptide Y (NPY), agouti-related peptide (AGRP), ghrelin, anorexigenic α-melanocyte-stimulating hormone (α-MSH), corticotropin-releasing hormone (CRH), and cocaine- and amphetamine -regulated transcript (CART) [1,2,3].

Neuromedin U (NMU) is a bioactive peptide that was first isolated from the porcine spinal cord and has a potent contractile effect on smooth muscle [4]. The sequence of NMU is highly conserved across species, implying that NMU has critical physiological functions. The NMU system, which includes the NMU-specific receptors, NMUR1 (also known as GPR66/FM-3) and NMUR2 (also called FM-4/TGR-1), which both belong to the G-protein-coupled receptors (GPCRs), is essential for the contraction of smooth muscles and is involved in feeding behavior, energy expenditure, stress responses, circadian rhythmicity, and inflammation [5,6,7,8,9,10,11,12]. Studies of genetic ablation or overexpression of NMU have revealed that there is crosstalk between this system and the pathogenesis of obesity [10,13]. In the last decade, there have been several reports that the NMU system has a variety of physiological roles related to obesity or obesity-related disorders in the central nervous system (CNS) and the peripheral tissues. This review provides an overview of these novel functions of the NMU system, including homeostatic or hedonic feeding behavior, obesity-related complications, insulin secretion, and inflammation, and summarizes each function in terms of the molecular aspects and therapeutic possibilities.

## 2. Biology of NMU and NMU-Related Peptides

### 2.1. Structure of NMU and Its Receptors

**Figure 1:**
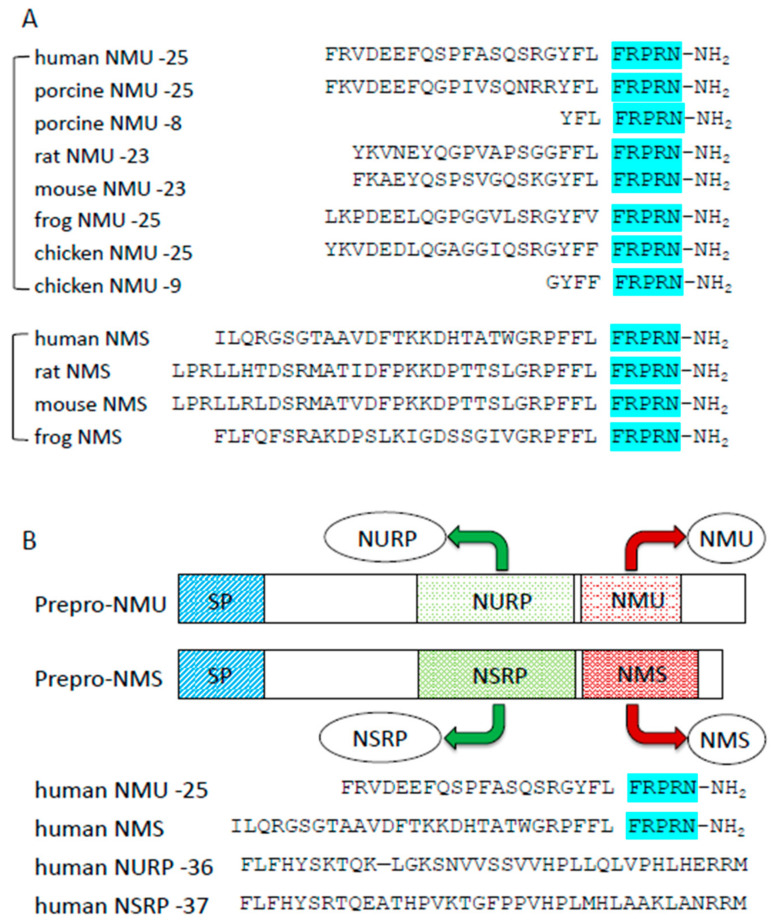
Structures of NMU and NMU-related peptides. (**A**) Comparison of amino acid sequences of NMU and NMS comparison in different species. The C-terminal sequences are highly conserved (highlighted). (**B**) Comparison of NMU, NMS, NURP, and NSRP.

Neuromedin U (NMU) was initially isolated from the porcine spinal cord and has potent smooth muscle contraction effects [4]. The sequence of NMU is highly conserved across species, implying that NMU has critical physiological functions. There are two major molecular forms: one is an extended form, NMU-23 or NMU-25, consisting of 23 or 25 amino acid peptides, respectively, and the other is a truncated 8- or 9-amino-acid C-terminal fragments, NMU-8 or NMU-9. The amidated C-terminal pentapeptide (-Phe-Arg-Pro-Arg-Asn-NH_2_) is totally conserved in most vertebrates, except goldfish [4,14,15,16,17,18,19,20,21,22,23] (Figure 1). In particular, the C-terminal heptapeptide is fully conserved in mammalian species. Amidation is necessary for receptor activation, because NMU-8 lacking an amide group does not induce intracellular Ca^2+^ influx via NMU receptors [24,25], and it does not cause uterine contraction or influence blood pressure [4,26]. It is also reported that Arg7 of NMU-8 is considered very important for receptor binding affinity or smooth muscle contraction activity, because these functions are abolished when this residue is replaced by others [25]. For decades, the specific receptors for NMU were not identified and the physiological functions of NMU were poorly understood. However, in 2000, several groups identified two specific receptors of NMU, NMUR1, and NMUR2, which were orphan GPCRs [5,6,24,27,28,29,30,31]. The gene for NMUR1 is located on human chromosome 2, and that for NMUR2 is found on chromosome 5. Both of these GPCRs, which possess a seven-transmembrane domain [25,27,32], and activation of NMUR1 or NMUR2 lead to the activation of Gaq/11 and Gai, but not Gas, resulting in intracellular Ca^2+^ elevation mediated by phospholipase C activation, as well as the inhibition of forskolin-induced cAMP accumulation [5,6,24,25,27,29,30]. Elevation of intracellular Ca^2+^ results in the release of arachidonic acid, which is thought to be mediated by Ca^2+^-dependent activation of phospholipase A2 [27,30]. The NMUR1 and NMUR2 receptors have some degree of specificity: for example, NMUR1 signals mainly via Gaq/11 and NMUR2 signals via Gai [33]. Receptors other than NMUR1 and NMUR2 could also interact with NMU, although their details have not yet been determined [34,35,36,37,38]. Further studies on whether NMU binds to such other receptors will be performed in the future.

### 2.2. Distribution of NMU

NMU-like immunoreactive cells (NMU-LI cells) are widely distributed but are found mainly localized in the gastrointestinal tract (GIT) and the CNS. In addition, low-level NMU-like immunoreactivity (NMU-LI) has been detected in the plasma or serum, suggesting that NMU is a neuropeptide acting as a local, rather than circulating, hormone [39]. Examination of the peripheral tissues has revealed large numbers of NMU-LI cells in the GIT (especially in the duodenum, jejunum, caecum, colon, and rectum), especially in Auerbach’s Meissner’s plexi of the GIT [40], and these cells are usually detected in the enteric nervous system [18,41,42,43,44]. Although NMU-LI cells are not localized in the nerve fibers projecting to the smooth muscle layers, there could be a high turnover of this peptide in these fibers [43,44]. NMU-LI cells coexist with different types of neurons, such as cholinergic, noncholinergic, and sensory neurons expressing calcitonin gene-related peptide, substance P, vasoactive intestinal peptide, or NPY, suggesting that NMU has multiple roles in the GIT [43,44]. NMU-LI cells can also be found in other peripheral regions, such as the genitourinary tract, and in the ureter, vas deferens, prostate, fallopian tubes, and urethra [39]. Other studies have detected NMU expression in the testis, ovary, thyroid gland, spleen, lymphocytes, adipose tissue, endothelial cells, keratinocytes, and placenta [12,24,27,29,45].

In the CNS, NMU-LI cells are widely distributed in both the spinal cord and brain. The brain regions with high levels of NMU-LI cells are the ACTH-releasing corticotrophs of the anterior pituitary gland. Moderate levels of NMU mRNA expression were detected in the striatum, hypothalamus, medulla oblongata, cingulate gyrus, and medial frontal gyrus [27]. Particularly in the hypothalamus, NMU mRNA is expressed in the arcuate nucleus (ARC) and suprachiasmatic nucleus (SCN) in mouse and rat. Furthermore, NMU mRNA is abundantly expressed in the dorsomedial hypothalamic nucleus (DMH) and ventromedial hypothalamus (VMH) in mouse but weakly expressed in the DMH in rat [46]. In contrast, low to moderate level of NMU-LI has been detected in the locus coeruleus, thalamus, hypothalamus, and substantial nigra [39]. In the spinal cord, greater numbers of NMU-LI cells are localized in the dorsal horn and dorsal root ganglia than in the ventral horn. This distribution pattern suggests NMU has a role in sensory activity [27,29,39].

### 2.3. NMU Receptor Distributions

Many reports have shown that NMUR1 is expressed mainly in the peripheral tissues–primarily in the GIT. In contrast, NMUR2 is expressed predominantly in the CNS. Human NMUR1 mRNA has been detected at high levels in the stomach and small intestine, with lower-level detection of NMUR1 mRNA in the pancreas, adrenal cortex, heart, lung, trachea, mammary gland, bone marrow, peripheral lymphocytes, genitourinary system, placenta, mammary gland, spleen, and adipose tissue [6,24,28,29,47]. In contrast, NMUR2 mRNA expression is restricted to the brain, including in the hypothalamus, hippocampus, substantia nigra, medulla oblongata, pontine reticular formation, spinal cord, thalamus, and cerebral cortex [5,6,31]. In situ hybridization studies revealed that NMUR2 mRNA was detected in the ARC and DMH, surrounding the VMH, the ependymal layer of the third ventricle and the paraventricular nucleus (PVN) of the hypothalamus [46]. NMUR2 mRNA has been found not only in the CNS but also in the peripheral tissues; it has been detected in the testis, GIT, genitourinary tract, liver, pancreas, adrenal gland, thyroid gland, lung, trachea, spleen, thymus, and testis [28,30,31,47].

### 2.4. Neuromedin S, Another Ligand of NMU Receptors

In 2005, Mori et al. used reverse pharmacological techniques to identify neuromedin S (NMS) as another endogenous ligand of NMURs [48]. NMS shares a C-terminal amino acid sequence with NMU (Figure 1). NMS was expressed mainly in the SCN of the hypothalamus. NMS has almost the same affinity for NMUR1 and NMUR2 as NMU, and the physiological functions of NMS are similar to those of NMU. Indeed, central NMS treatment of rats or mice results in decreased food intake, increased energy expenditure, activation of sympathetic activity, and regulation of circadian rhythmicity. Although the NMU and NMS genes are located on different chromosomes, the primary structures of both precursors are very similar. In 2017, the precursors of NMU and NMS were found to produce other peptides distinct from NMU and NMS; they were named NURP (neuromedin U precursor-related peptide) and NSRP (neuromedin S precursor-related peptide) [49] (Figure 1). Recently, it was reported that NURP and NSRP had some effects on sympathetic nerve activity, prolactin releasing thermogenesis, and regulation of locomotor activity, but not on food intake; however, their effects differ from those of NMU and NMS [50,51]. Further studies are needed to clarify these phenomena.

## 3. Homeostatic Regulation of Feeding Behavior

It is well known that hypothalamic and peripheral neuropeptides are involved in the regulation of energy homeostasis. The distributions of NMU receptors suggest that it is highly probable that NMU regulates the feeding behavior and energy metabolism mediated by hypothalamic neuronal circuits. In particular, NMU is produced mainly in the ARC of the hypothalamus, which is a master brain region for regulating appetite and energy homeostasis [2]. NMUR2, one of the cognate receptors for NMU, exists in the PVN of the hypothalamus [6]. Several reports have demonstrated that intracerebroventricular (ICV) injection of NMU in rats or mice leads to anti-obesity phenotypes with decreased food intake and body weight and increased physical activity, energy expenditure, and non-exercise-related thermogenesis [5,6,7,8,52,53]. In contrast, central injection of anti-NMU IgG in rats increases food intake [5], and fasting reduces NMU expression levels in the VMH [6]. In addition, transgenic mice ubiquitously overexpressing NMU are hypophagic and leaner than wild-type mice, even when fed a high-fat diet [13]. They also show high insulin sensitivity and elevated mRNA levels of hypothalamic AGRP, NPY, proopiomelanocortin (POMC), and melanin-concentrating hormone (MCH). In contrast, NMU gene-deficient mice (NMU knockout [KO] mice) exhibit hyperphagia, increased adiposity, and reduced locomotor activity and energy expenditure, and they develop obesity with late-onset hyperlipidemia and fatty liver [10]. Moreover, this obese phenotype in NMU KO mice is abolished by central NMU administration. ICV injection of NMU in rodents increases the abundance of c-fos-expressing neurons, indicating neuronal activation, in the PVN, ARC, supraoptic nucleus (SON), DMH, lateral hypothalamic area (LHA), and amygdala, and the parabrachial nucleus, nucleus tractus solitarius (NTS), and ventrolateral medulla of the brain stem [54]. In addition, upon ICV administration of NMU to rats, c-fos is co-expressed with CRH in neurons in the PVN, and CRH mRNA levels are upregulated in the PVN [55]. As NMUR2 is expressed in the PVN, direct microinjection of NMU into the PVN region in rats results in reduced food intake and increased activity levels. Furthermore, administration of a specific NMUR2 agonist, EUK2010, decreases body weight in rodents [56]. Although NMUR2-null mice do not have an obvious obese phenotype, these mice show a reduction in body weights with central NMU injection [34,57]. In contrast, NMU KO mice have decreased CRH mRNA expression levels in the PVN, and NMU anorexigenic effects, such as decreased food intake, increased locomotor activity, and increased body temperature and energy expenditure, are abolished in CRH KO mice [8,10]. This evidence suggests that NMU is an essential anorexigenic peptide mediated via the NMUR2 pathway. It also suggests that CRH in the PVN is one of the main targets of central NMU anorexigenic function. Recently, Takayama et al. reported that intra nasal treatment of NMUR2 selective agonist (CPN-116, CPN-221) decreased body weight and food intake in mice in a dose-dependent manner [58,59]. In this way, NMUR2 antagonists are potential candidates for anti-obesity drugs in humans.

Many appetite-regulating peptides are closely related to leptin signaling pathways in the hypothalamic circuit and have some roles in feeding behavior and energy homeostasis under the control of leptin [1,2]. Leptin is a potent anorexigenic hormone produced in adipocytes and activates neuronal cells in the ARC and PVN of the hypothalamus, which is the brain region containing orexigenic peptides such as NPY and AGRP and anorexigenic peptides such as α-MSH and CRH [3,60,61]. There have been some reports that leptin has a role in regulating NMU production in the hypothalamus [6,62]. In general, obese mice, as well as obese humans, demonstrate hyperleptinemia and resistance to exogenous leptin treatment; however, obese NMU KO mice have normal sensitivity to exogenous leptin [10,63,64,65,66], and anorexigenic effects of NMU remain in leptin-deficient ob/ob mice or leptin- receptor-deficient db/db mice [10]. These findings imply that NMU and leptin signaling pathways in the hypothalamus are independent in energy homeostasis. In contrast, Jethwa et al. reported that hypothalamic NMU may have a stimulatory action on the hypothalamo-pituitary-adrenal (HPA) axis, which is partially mediated by leptin-induced HPA activation [67]. Thus, the relationship between the NMU system and leptin signaling pathway is still conflicting and remains to be studied.

NMU also has peripheral roles in controlling feeding behavior and energy homeostasis [68]. Peripheral acute or chronic NMU administration reduces food intake and body weight, and increases the body temperature, metabolic rate, and plasma levels of the anorectic peptides glucagon-like peptide 1 (GLP-1) and peptide tyrosine-tyrosine (PYY) in mice [68]. These actions are mediated by the NMUR1 pathway, because they are abolished in NMU R1 KO mice [68]. As NMU has not been much found to circulate at detectable levels in the plasma, these peripheral actions are thought to be a local action of NMU [39]. This evidence suggests that a stable NMUR1 agonist for peripheral administration could be a potential candidate for treating obesity and diabetes.

Not only in rodents but also in humans, NMU has a tight connection with obesity [69]. Hainerova et al. identified two variants of NMU in humans. One variant is NMUAla19Glu polymorphism, an amino acid change, located in the signal peptide of the pre-pro-NMU, which is important for cellular translocation. This polymorphism is believed to reduce export of the peptide and has a tight connection to an obese phenotype in middle-aged Caucasians. Another is the rare NMU Arg165Trp mutation found in a Czech family. It has autosomal dominant inheritance, is located in active NMU peptides, and leads to hypertriglyceridemia and childhood-onset obesity [69].

These findings clearly demonstrate that NMU plays a crucial role in feeding behavior and energy homeostasis in both rodents and humans.

## 4. The Brain Reward System and Stress, and Their Relationships with the NMU System

Feeding behavior is regulated by not only homeostatic controls but also hedonic and stress controls. Homeostatic feeding behavior is based on the energy needs of the body, but hedonic feeding behavior is tightly related to the brain reward system, which leads to pleasure [70]. Recent reports have shown a powerful link between feeding behavior and brain reward systems. Obese NMU-KO mice show a “binge-like eating” phenotype that is closely associated with the brain reward system [10,70,71]. Although Egecioglu et al. reported that NMUR2 KO mice fed a high fat diet have increased body weight [72], Peier et al. reported that NMUR2 KO mice have decreased bodyweight regardless of the type of diet [73]. There were some discrepancies in these reports, and the mechanisms of NMU-NMUR2 feeding behaviors were not clearly defined. However, NMUR2 KO mice might prefer certain diets, such as a high fat diet, but not others. In 2014, Benzon et al. demonstrated that NMU-NMUR2 signaling in the hypothalamic PVN regulated consumption of, and preference for, a high fat diet [74]. They established conditional knockdown of NMUR2 in the PVN of adult rats by using adeno-associated-virus (AAV)-mediated RNA interference; the AAV was engineered to express a small hairpin RNA (shRNA) designed to knock down NMUR2 (shNMUR2) [74]. First, they demonstrated that rats with shNMUR2 in the PVN showed increased food intake of a high fat diet but not standard chow, implying that NMU-NMUR2 signaling in the PVN influences the intake of obesogenic food. In addition, rat with shNMUR2 in the PVN gained more body weight when given ad libitum access to the high fat diet, but there was no effect on their body weight on standard chow. Moreover, shNMUR2 rats showed no preference for a sucrose diet, which also contained reinforcing components of food. Some reports have found that dietary fat is more obesogenic than carbohydrates. Signaling mediated by receptors that do not involve NMUR2 (e.g., the μ-opioid receptor) also has a critical role in preference for carbohydrates such as sugars [74,75]. Furthermore, shNMUR2 rats show increased binge-like eating behavior. Binge-like eating is mediated via dopamine, which is released into the nucleus accumbens (NAc) during repeated binge eating behavior [74,76]. On the basis of this evidence, Benzon et al. concluded that decreased NMUR2 signaling in the PVN of adult rats promotes the palatability of obesogenic food and increases body weight [74].

It has been reported that hedonic eating and drug abuse share common functional mechanisms mediated by the brain reward system via the mesolimbic dopamine system [70]. In the last decade, it has also been reported that many appetite-regulating peptides, such as ghrelin, NPY, AGRP, POMC, and GLP-1, have essential roles in the progress of drug addiction, obesogenic food preference or in brain reward systems [77,78,79]. Several studies have shown the link between the NMU system and the brain reward system [77,80,81,82,83,84]. In particular, the NMU-NMUR2 pathway has an important role in regulating the reinforcement value of not only preference for obesogenic, palatable food [74,84] but also amphetamine-evoked locomotion [82] and alcohol [81]. NMU and NMUR2 are highly expressed in the area associated with the brain reward system, such as the NAc [77]. Anan et al. reported that some cocaine-induced c-fos-expressing neurons were co-localized with NMU-immunoreactive neurons in the NAc, the caudate putamen (CPu) and the basolateral amygdala (BLA), which are key brain regions associated with the brain reward system [85]. Not only in rodents, but also in humans, a single nucleotide polymorphism in NMUR2 was associated with alcohol abuse in a genome-wide allelic association study [83]. This evidence indicates that the NMU system plays an important role in reward processes, but further studies are needed to elucidate the detailed mechanisms of the NMU system in this field.

It is also well known that stress responses affect feeding behavior. Central NMU administration not only reduces food intake but also elicits a stress response via mobilization of CRH and activation of the HPA axis [8]. Acute central administration of NMU induces excessive grooming behavior, which represents a stress response [9] as do increase locomotor activity [7,9] and elevation of systemic corticosterone levels [62]. In contrast, acute peripheral NMU treatment does not elicit excessive grooming behavior or increased locomotor activity, nor does it elevate systemic corticosterone when administered chronically [68]. As mentioned, the NMU system also has a role in stress-induced feeding behavior.

Thus, the relationship of the NMU system and feeding behavior in relation to the mood has not been fully elucidated, and further studies of the detailed mechanisms are required.

## 5. NMU as a Modulator of Other Metabolic Disorders

As mentioned above, the NMU system is definitely a key player in the regulation of feeding behavior and energy metabolism. Recent studies have shown that the etiology of obesity is not limited to the disruption of energy homeostasis but is also intimately associated with inflammation. There are now many reports of the tight connections between obesity and type 2 diabetes and complications such as cardiovascular diseases, including hypertension or atherosclerosis, chronic kidney disease, and fatty liver disease [86,87]. However, it has not been elucidated whether the NMU system is directly involved in obesity-related complications such as non-alcoholic fatty liver disease (NAFLD) and non-alcoholic steatohepatitis (NASH). NAFLD is a hepatic complication of obesity, type 2 diabetes, and hyperlipidemia; it is one of the most common liver disorders and is characterized by the accumulation of lipids in hepatocytes [88,89,90]. Simple hepatic steatosis progresses to NASH with chronic inflammation of the liver and fibrosis; this then advances to liver cirrhosis or hepatocellular carcinoma. These days, the accepted hypothesis of the pathophysiological progress of NASH is the “multiple parallel hits” hypothesis [91]. Teranishi et al. did not detect the expression of NMU and NMUR2 mRNAs in the livers of mice on a normal diet or high fat diet. NMUR1 mRNA was detected in the liver tissues of mice, but there was no difference in the expression levels of NMUR1 mRNA between mice on a normal diet and those on a high fat diet [92]. In contrast, Teranishi et al. demonstrated that NMU mRNA levels were upregulated in the NASH model, which was confirmed by serology and histology, in KKAy mice (a cross between diabetic KK and lethal yellow (Ay) mice) on a CDAA (choline-deficient and iron-supplemented L-amino acid-defined) diet. Furthermore, NMUR1 mRNA levels were also significantly greater in the NASH livers than in normal liver tissues. Immunohistochemical examination detected positivity for NMU-immunoreactivity in the macrophage population in the liver. Thus, NMU and NMUR1 production was activated in the NASH liver and depended on the progression of NASH. In addition, in a unique mouse model, NMU overproduction in the liver was induced by hydrodynamic injection of DNA (also known as Sleeping Beauty transposon system) and the mice were fed a CDAA diet. They showed accelerated to production of proinflammatory cytokines, such as interleukin (IL)-6, IL-1β, and markers of fibrosis such as monocyte chemoattractant protein-1(MCP-1), α-smooth muscle actin (α-SMA) in the liver. This then exacerbated NASH pathogenesis in the mice. However, there were no changes in expression levels of the anti-inflammatory cytokines IL-4, IL-10, and IL-13 in the livers of these NMU-Sleeping Beauty mice [92]. Thus, manipulation of the NMU system provides new insights into the molecular mechanisms of NASH. Currently, there are no approved pharmacological interventions or approved biomarkers for the diagnosis of NAFLD/NASH. However, the above evidence suggests that the NMU system could be one of the potential candidates for the development of a biomarker for detecting NASH progression.

## 6. Insulin Secretion

Another intriguing role of the NMU system—in glucose homeostasis—has been reported [68,93]. NMU and NMUR1 mRNAs are expressed in the pancreatic islets of the rat. Moreover, NMUR1 mRNA and protein have been detected in human pancreatic β-cells, and NMU treatment of isolated human islets suppresses insulin secretion [94]. In addition, the peripheral treatment with NMU results in elevated GLP-1 levels in mice and improved glucose tolerance via NMUR1 signaling [68]. These NMU functions of glucose homeostasis are mediated by NMUR1, because they are absent in NMUR1-deficient mice [68]. This evidence implies that the NMU/NMUR1 system has a physiological role as incretin in the pancreas. On the other hand, Zhang et al. reported that NMU has an opposite physiological function in insulin secretion and glucose metabolism [90]. NMU is also expressed in insulin secreting β-cells in mice and in β-cell-derived MIN6-K8 cells but is not co-localized in somatostatin- or glucagon-secreting cells in the pancreas. Not only NMU mRNA, but also NMUR1 mRNA is expressed in mouse pancreatic islet and MIN6-K8 cells, whereas NMUR2 mRNA is not detectable in either. NMU clearly decreases glucose-stimulated insulin secretion (GSIS) in MIN6-K8 cells and isolated pancreatic islets in the mouse. In addition, a partial agonist of NMUR1 (6a) also attenuates GSIS in both mouse islets and MIN6-K8 cells [93,95]. In in vivo mouse experiments, NMU administration has significantly increased blood glucose levels compared with those in saline-administrated mice. In contrast, NMU treatment attenuates glucose-stimulated insulin secretion and the average insulin incremental area under the curve of plasma insulin (iAUC). Calcium influx is a key factor in the regulation of GSIS, and NMU suppressed the intracellular Ca^2+^ influx in MIN6-K8 cells, which expresses NMUR1. Anti-NMU IgG treatment of the isolated mouse pancreatic islets and knockdown of NMU by *siNmu*-transfected MIN6-K8 cells clearly showed increase in insulin secretion. Furthermore, *siNmu* RNAs significantly reduces NMU mRNA expression in MIN6-K8 cells. Although *siNmu* in MIN6-K8 cells leads to GSIS elevation with suppression of [Ca^2+^]i, Nmu knockdown has no effect on the insulin content of MIN6-K8 cells. Notably, NMU has no effect on intracellular cAMP levels upon forskolin stimulation and does not completely abolish the GSIS stimulated by Epac2 selective activator. In contrast, however, subcutaneous NMU administration improves glucose tolerance by increasing insulin secretion in mice with diet-induced obesity [68]. The reason for this phenomenon might be the elevation of plasma GLP-1, although the mechanism of NMU-induced elevation of plasma GLP-1 in obese mice is not clear [96]. Thus, the overall effect of the NMU-NMUR1 system is that of decretin, attenuating GSIS in an autocrine or paracrine fashion. On the other hand, a study in NMU/NMS double knockout (dKO) mice showed that plasma insulin levels did not differ between NMU/NMS dKO and wild-type mice with or without glucose injection. In addition, NMU treatment of NMU/NMS dKO and wild-type mice had no significant effects on the plasma glucose or insulin levels [97]. In vitro and in vivo experimental data show that the NMU-NMUR1 system is a key pathway for suppressing insulin secretion in local pancreatic β-cells, although its systemic involvement is not yet clear.

## 7. Inflammation

Recently, it was reported that low-grade inflammation—also called metainflammation—is a contributor to the pathogenesis of obesity and obesity-related diseases [86]. In the steady state, adipose tissue maintains metabolic homeostasis mediated by adipocytes and tissue-resident immune cells, particularly macrophages in adipose tissue [86,98]. In contrast, a high fat diet induces an increase in adipocyte numbers and excessive adipocyte hypertrophy, and leads to acute adipose tissue inflammation. This, in turn, contributes to adipose tissue remodeling and further recruits monocytes and exacerbating inflammation [86,99]. This loop of sustained inflammation leads to cellular or systemic insulin resistance and also permeates into other tissues such as the liver, pancreas, muscles, and brain.

NMU and its receptors, NMUR1 and NMUR2, are also expressed by some immune cells. NMU mRNA is detectable in antigen-presenting cells, including monocytes and dendritic cells. NMUR1 mRNA has been detected in T cells and natural killer cells and is detected slightly in hematopoietic cells, such as eosinophils and mast cells [24,33,100,101]. These distribution patterns of NMU and NMUR1 suggest that they have a role in immune responses. NMU was initially been reported to induce the release of some kinds of cytokines such as interleukin-4 (IL-4), IL-5, IL-6, IL-10, and IL-13 via NMUR1 dependence in T-helper 2 cell lines [102]. IL-6 secretion is reduced in the macrophages of NMU-deficient mice, suggesting that NMU promotes IL-6 secretion and inflammation [35,101]. Moreover, activation of the NMU-NMUR1 cascade in mast cells induces degranulation and neutrophil infiltration [12]. NMU also promotes eosinophil activation, migration, and adhesion, mediated by the NMUR1 signaling pathway, at local inflammatory sites, suggesting that NMU has some effects on allergic reactions [100]. However, the exact functions of the NMU system in regulating cytokine production are still unknown.

Intriguing recent reports have revealed that NMU enhances type 2 innate-lymphoid-cell (ILC-2)-driven asthma and allergic lung inflammation [103,104,105]. ILC-2s are effector cells that mainly regulate immune responses at the airway barrier surface [106]; and activated ILC2s produces type 2 cytokines such as IL-13 to induce allergic inflammation at mucosal surfaces [107,108]. There is much epidemiological evidence of the association between asthma and obesity, but the exact mechanisms and pathogenesis are not yet defined [109]. Together with NMUR1, which is expressed in ILC2s, NMU is a crucial modulator of both obesity and asthma pathogenesis; further studies could provide some new mechanisms mediated by the NMU system in the pathogenesis of these disorders. The NMU system therefore has important roles in modulating various kinds of immune responses.

## 8. Conclusions

**Figure 2:**
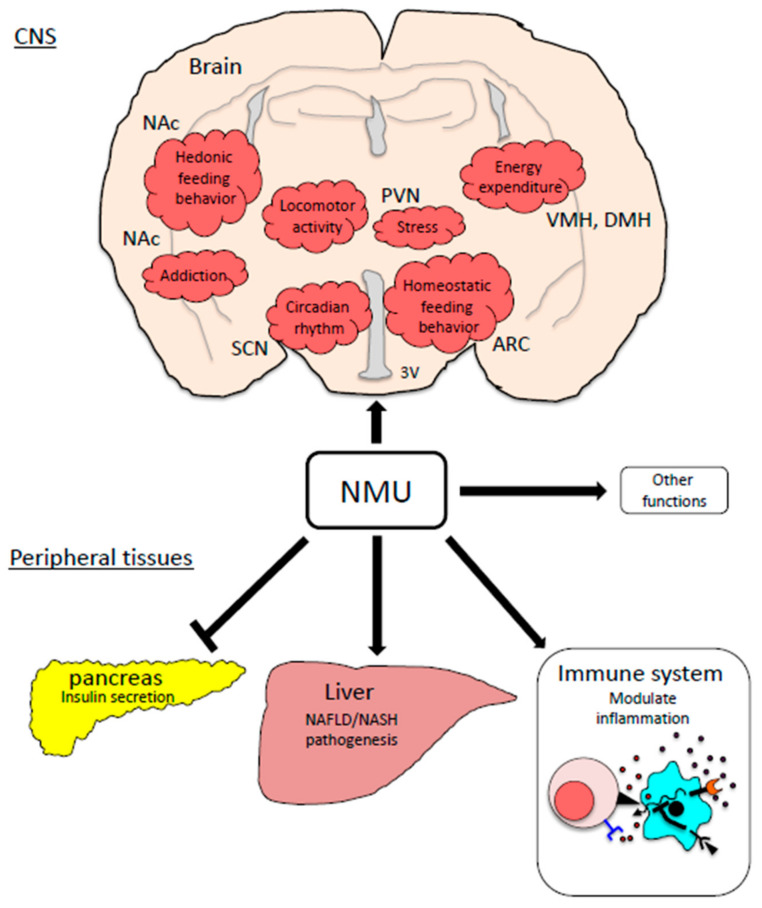
Physiological functions of the NMU system related with obesity pathogenesis. ARC: the arcuate nucleus, PVN: the paraventricular nucleus, DMH: the dorsomedial hypothalamic nucleus, VMH: the ventromedial hypothalamus, NAc: the nucleus accumbens, SCN: the suprachiasmatic nucleus.

NMU is a multifunctional neuropeptide with pleiotropic roles, including gastrointestinal motility, energy homeostasis, stress response, circadian rhythmicity, cardiovascular function, and immune modulation. In the last decade, several reports have indicated that the NMU system plays important roles in obesity and other metabolic disorders (Figure 2). Even though the molecular mechanisms underlying these roles are not yet fully defined, the new biological functions of the NMU system, as summarized in this review, are relevant to the etiology of various aspects of obesity and other metabolic disorders and are thus important in different pathological conditions. Further studies are needed to understand in depth the molecular mechanisms of each of these functions. The resulting findings will lead to novel therapeutic possibilities for the NMU system as the target of anti-obesity drug development.

## Data Availability

Not applicable.
